# Inhibition Effect of *Pseudomonas stutzeri* on the Corrosion of X70 Pipeline Steel Caused by Sulfate-Reducing Bacteria

**DOI:** 10.3390/ma16072896

**Published:** 2023-04-05

**Authors:** Lina Qiu, Dandan Zhao, Shujia Zheng, Aijun Gong, Zhipeng Liu, Yiran Su, Ziyi Liu

**Affiliations:** 1 School of Chemistry and Biological Engineering, University of Science and Technology Beijing, Beijing 100083, China; 2 Beijing Key Laboratory for Science and Application of Functional Molecular and Crystalline Materials, University of Science and Technology Beijing, Beijing 100083, China

**Keywords:** microbiologically influenced corrosion inhibition, sulfate-reducing bacteria, *Pseudomonas stutzeri*, X70 steel

## Abstract

Microbiologically influenced corrosion (MIC) is a common phenomenon in water treatment, shipping, construction, marine and other industries. Sulfate-reducing bacteria (SRB) often lead to MIC. In this paper, a strain of *Pseudomonas stutzeri* (*P. stutzeri*) with the ability to inhibit SRB corrosion is isolated from the soil through enrichment culture. *P. stutzeri* is a short, rod-shaped, white and transparent colony with denitrification ability. Our 16SrDNA sequencing results verify the properties of *P. stutzeri* strains. The growth conditions of *P. stutzeri* bacteria and SRB are similar, and the optimal culture conditions are about 30 °C, pH 7, and the stable stage is reached in about seven days. The bacteria can coexist in the same growth environment. Using the weight loss method, electrochemical experiments and composition analysis techniques we found that *P. stutzeri* can inhibit the corrosion of X70 steel by SRB at 20~40 °C, pH 6~8. Furthermore, long-term tests at 3, 6 and 9 months reveal that *P. stutzeri* can effectively inhibit the corrosion of X70 steel caused by SRB.

## 1. Introduction

Metal corrosion is a spontaneous chemical, electrochemical or biological process that destroys metal and its physical and chemical properties [1]. It affects global security in areas as diverse as oil and gas transportation, offshore engineering equipment and water treatment [2,3,4]. Among all kinds of metal corrosion, microbially induced corrosion (MIC) accounts for about 20% of the total corrosion [5], and can cause economic losses of tens of billions of dollars every year [6]. Many large accidents have been directly or indirectly caused by MIC, such as the fire on the offshore drilling platform of Mexican oil giant PEMEX in 2015 [7], the Prudhoe Bay oil spill on the North Slope of Alaska [8] and a large explosion caused by a natural gas pipeline leak in Carlsbad, New Mexico [9]. Many types of microorganisms are involved in MIC, which can be divided into aerobic, anaerobic and facultative, according to different requirements for oxygen. These microorganisms generally form biofilms on the surface of metal materials simultaneously. Aerobic and facultative bacteria can consume O_2_ in the system during their growth and provide local anaerobic growth conditions for anaerobic bacteria while growing [10]. Common corrosive microorganisms include bacteria, archaea and fungi, such as sulfate-reducing bacteria (SRB) [11], sulfate-reducing archaea (SRA) [12], nitrate-reducing bacteria (NRB) [13], methanogenic bacteria [14], acid-producing bacteria (APB) [15], iron-reducing bacteria (IRB) [16], iron-oxidizing bacteria (IOB) [17], sulfur-oxidizing bacteria (SOB) [18], manganese-oxidizing bacteria (MOB) [19], a variety fungi [20] etc. Because sulfate is widely distributed in many systems, such as seawater, brackish water and agricultural runoff water [21], SRB have been extensively studied for decades as major pathogenic microorganisms in microbial systems. Traditional anti-corrosion measures such as coating, surface treatment and corrosion inhibitors can control most corrosion problems, but inevitably bring environmental problems because of the accumulated release of toxic substances [6,22,23].

Microbiologically influenced corrosion inhibition (MICI) is caused by microorganisms’ direct or indirect actions [6]. Compared with traditional anti-corrosion methods, MICI is characterized by low cost, environmental friendliness as well as moderate application and maintenance requirements. Therefore, it has ecological and economic significance and will become the development direction of the next generation of preservative technology. However, due to the diversity of microorganisms and the complexity of their metabolic processes influenced by environmental factors, MICI is still facing the challenge of practical application. Therefore, the research on MICI in China and abroad is still in the initial stage. In addition, some problems remain to be solved, such as single species and a lack of systematic understanding of the preservative mechanism.

*Pseudomonas stutzeri* is a Gram-negative facultative anaerobic bacterium with strong denitrification activity and high denitrification capacity. It is widely used in biological denitrification [24,25,26,27]. Liu et al. showed that the inhibition of corrosion of deposit-covered X80 pipeline steel was due to *P. stutzeri* in seawater containing CO_2_ [28]. Fu et al. studied the effects of *P. stutzeri* on the biocorrosion of X80 pipeline steel for different nitrate and nitrite concentrations [29]. In this work, a strain of *P. stutzeri* with SRB corrosion resistance was isolated and purified from the soil by enrichment culture, and the anti-corrosion behavior of the bacterium against X70 steel in the presence of SRB was investigated. This provides a theoretical basis for the further development of new green, low-cost and long-term novel SRB corrosion inhibition technologies.

## 2. Materials and Methods

### 2.1. Isolation and Identification of P. stutzeri

The liquid culture medium used for *P. stutzeri* was as follows (g/L) [25,30]: K_2_HPO_4_ (2.0), NH_4_Cl (0.5), Na_2_S_2_O_3_·5H_2_O (5.0), MgSO_4·_7H_2_O (0.6), FeSO_4_·7H_2_O (0.01), KNO_3_ (2.0), NaHCO_3_ (1.0), NaKC_4_H_4_O_6_ (20.0) and pH 1.0~7.2. Before inoculation, the liquid medium was sterilized by autoclaving at 120 °C for 20 min. Then, 1.5–2% agar was added to the solid medium.

The soil sample was weighed to 10 g, inoculated into a conical flask containing 100 mL liquid medium, and shaken for 10 min so that the soil sample was well-mixed and incubated at a constant temperature of 30 °C. After the upper layer of liquid was turbid, 5 mL of the upper layer of clear liquid was inoculated into 50 mL of the liquid medium in the sterile table, mixed well and continued to incubate at a constant temperature. When the bacterial fluid became turbid again, 0.2, 0.5, 1.0, 1.5 and 2.0 mL of bacterial fluid were taken to the sterile table, quickly added to sterilized Petri dishes and poured into sterilized warm solid media (the temperature was below 60 °C). The media and the bacterial solutions were mixed well by gentle shaking. After cooling and solidifying, the plates were inverted into the constant-temperature incubator at 30 °C for observation. After 7 days of incubation, scattered single colonies appeared on the surface and inside of the plates, and single colonies were separately picked and inoculated onto solid plates to continue the culture of the purified strains.

The single purified colonies were inoculated into sterilized test tubes with 10 mL of liquid medium and incubated at 30 °C. Once the bacterial solutions were turbid, the strains were preserved and set aside.

The identification of the strains was determined by microscopic observation, Giltay medium denitrification ability identification and 16S rDNA sequencing.

### 2.2. Growth Characteristics Experiment

The growth of bacteria has a greater impact on corrosion and anti-corrosion, and the corrosion effect of the strain is more evident at higher concentrations. For this experiment, the optical density at 600 nm (OD_600_) values of the experimental strains under different conditions of time, temperature and pH were studied by using a spectrophotometer to determine the growth characteristics of the strains.

1. Effect of temperature on the growth of SRB and *P. stutzeri*

SRB and *P. stutzeri* strains were inoculated in a liquid medium and incubated anaerobically at 10 °C, 20 °C, 30 °C, 40 °C and 50 °C for 7 days. The OD_600_ values at each temperature were measured to determine the optimal growth temperature of the strains.

2. Effect of pH on the growth of SRB and *P. stutzeri*

The SRB and *P. stutzeri* strains were inoculated equally in a liquid medium at pH 1.0, 3.0, 5.0, 7.0, 9.0, 11.0 and 13.0 for 7 days. The OD_600_ values were measured at each pH to determine the optimal growth pH of the strains.

3. Graphical growth curves of *P. stutzeri*, SRB and co-culture of *P. stutzeri* + SRB

The media of *P. stutzeri* and SRB were prepared separately, as well as the mixed medium mixed at a ratio of 1:1. *P. stutzeri*, SRB, as well as the mixture of SRB and *P. stutzeri* were inoculated in a 1/100 ratio separately and incubated anaerobically in blue-capped culture flasks. Then the OD_600_ values were measured by sampling every 24 h.

All experiments were implemented in three replicates and results were based on the mean after three replicates with a standard deviation range of 0.5–5.0%.

### 2.3. Exposure Experiments

#### 2.3.1. EDS and Weight Loss Measurement

Before soaking the specimens, holes were punched at the top middle, and each side was sanded to mirror-smooth using SiC sandpaper from 400 to 800, 1000 and 2000 mesh. Then, the specimens were washed with deionized water and anhydrous ethanol, blown dry and weighed and irradiated with UV light for at least 30 min. The steel sheets were suspended vertically inside the frosted jars with waterproof wires. The surface of the liquid was covered with liquid paraffin. After nitrogen was flushed into the bottle, the gap between the stopper and the bottle body was sealed with the sealing film to isolate the air. The experiments were divided into blank, SRB and SRB + *P. stutzeri*, with different control of incubation time, temperature, pH etc. All experiments were done three times in parallel. When the immersion time was reached, the steel sheets were removed and the surface was descaled according to the Chinese Standard “Corrosion of metals and alloys-Removal of corrosion products from corrosion test specimens” GB/T16545-2015.

The rust remover comprised 500 mL hydrochloric acid (ρ = 1.19 g/mL), 500 mL deionized water and 3.5 g of hexamethylene tetramine, well-mixed. After rust removal, samples were rinsed with deionized water and anhydrous ethanol, then dried and weighed. The inhibition effect of *P. stutzeri* bacteria on the corrosion caused by SRB under each condition was analyzed by the weight loss of steel sheets. The additional specimens were removed after soaking for 7, 14 and 21 days, before scanning X-ray energy-dispersive spectrometer (EDS, from Oxford Instruments, Abingdon, UK). For analysis of corrosion products, the specimens were pretreated by soaking in a phosphate-buffered saline solution containing 2.5% (*v/v*) glutaraldehyde for 8 h to immobilize cells. The specimens were then dehydrated using a serial dilution of ethanol (30%, 50%, 70%, 90% *v/v*), each for 15 min, except the final step for 30 min [31].

#### 2.3.2. Electrochemical Measurements

The steel sheets were polished to smooth grade by grade, soldered with copper conductor, then put into the mold and sealed with epoxy resin, with ethylenediamine as the curing agent. Then, the samples were reserved with a working surface of 10 mm × 10 mm, polished with sandpaper in steps to 2000 mesh before use, and then polished with w 2.5 and w 0.5 diamond grinding paste in turn, rinsed with deionized water, cleaned with anhydrous ethanol, sterilized under UV for 30 min and set aside. A three-electrode system was used for the electrochemical tests, with X70 steel specimen as the working electrode, platinum sheet as the auxiliary electrode, and Saturated Calomel Electrode (SCE) as the reference electrode, using a Chi760e electrochemical workstation. Specimens were divided into two experimental groups using the co-culture medium—one group was inoculated with SRB only and the other group was inoculated with SRB + *P. stutzeri*. For every 100 mL of medium, 10 mL of bacterial solution was inoculated. The test scan potential range was ±0.5 V vs. OCP, and the potential scan rate was 2 mV·s^−1^. The polarization curve (Tafel curve) of the steel sheet was measured. It is worth noting that potential scan rate has an essential role in minimizing the effects of distortion in Tafel slopes and corrosion current density analyses, as previously reported [32,33,34]. However, based on these reports, the adopted 2 mV/s has no deleterious effects on those Tafel extrapolations [32,33] to determine the corrosion current densities (i_corr_) of the examined samples.

### 2.4. Long-Term Corrosion Experiments

Initially, nine plastic reagent bottles (500 mL) were placed in 500 mL co-culture medium and autoclaved for 30 min. The coupons of X70 pipeline steel (18.7 mm × 7.8 mm × 1 mm) were provided by School of Materials Science and Engineering, USTB. Three experimental groups were set up, and the inocula are shown in Table 1. The samples were sealed and placed at room temperature for 3, 6 and 9 months to observe the corrosion and corrosion inhibition over a long period of time.

## 3. Results and Discussion

### 3.1. Identification of P. stutzeri Strain

#### 3.1.1. Observation of Colonies

After the collected soil samples were cultured in liquid and enriched several times with strains, the samples were isolated and purified by the spread plate method. The growth rate of colonies in the autotrophic medium was slow, and small beige or white colonies appeared on the plates after about 5 days of incubation. After 7 days of incubation, there was a significant difference between each soil sample plate’s surface and internal colony morphology. The small yellowish translucent needle-tip colonies on the surface of the plates were *P. stutzeri*.

**Figure 1:**
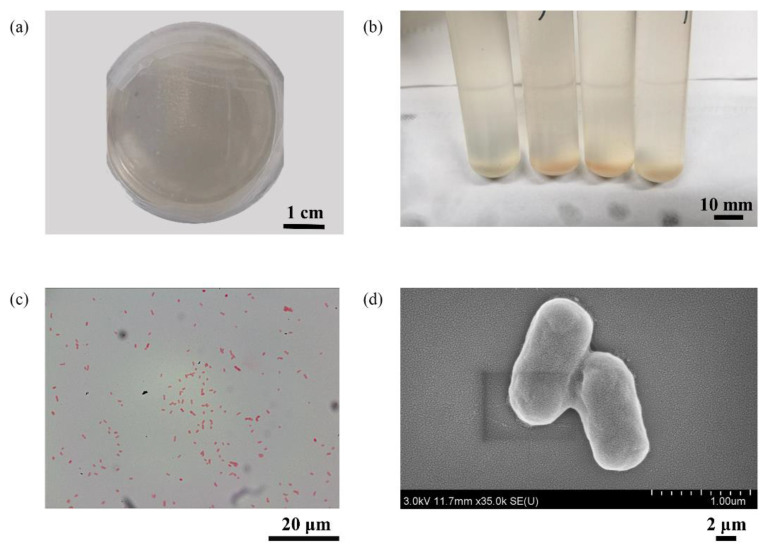
*P. stutzeri* colony (**a**); *P. stutzeri* liquid culture (**b**); *P. stutzeri* Gram staining (**c**); *P. stutzeri* SEM (**d**).

The single colonies of *P. stutzeri* were inoculated in the sterilized medium for more than three generations of enrichment. The acceleration voltage of SEM test was set to 3 kV and the emission current was set to 100 μA. Then, the purified strains were subjected to Gram staining and SEM observation, as shown in Figure 1. *P*. *stutzeri* stained dark red, indicating that they were Gram-negative bacteria, and the cells were short rods with a length between 0.5 and 1 µm. The Gram staining experiment also showed that the purified strains were relatively pure, and no other miscellaneous bacteria were mixed in.

#### 3.1.2. Identification of the Denitrification Capacity of *P. stutzeri*

**Figure 2:**
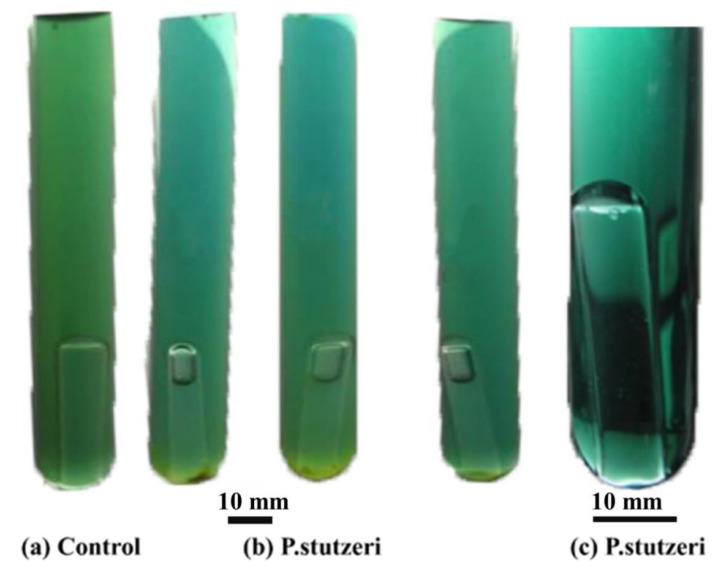
Identification of denitrifying bacteria using Giltay medium. (**a**) The blank Giltay medium; (**b**) medium for inoculation with *P. stutzeri*; (**c**) local amplification of (**b**).

After incubation with the Giltay medium, *P. stutzeri* could be observed for its discoloration reaction. *P. stutzeri* can turn the color of Giltay liquid medium blue-green as they are autotrophic denitrifying bacteria. Under anaerobic conditions, *P. stutzeri* can produce nitrogen, so air bubbles (N_2_) can be trapped in the Duchenne tubules. Without the presence of the denitrifying bacteria *P. stutzeri*, the Giltay medium was still dark green, but no air bubbles appeared in the Duchenne tubules. The results of the experiment are shown in Figure 2. *P. stutzeri* bacteria discolored the Giltay medium, and thus had a denitrification ability that could be used for subsequent experiments.

#### 3.1.3. Identification by 16S rDNA Sequencing

The sequencing of the strains was conducted by Sangon Biotech (Shanghai) Co., Ltd. (Shanghai, China). Then, the sequencing results were spliced with ContigExpress and the faulty parts at both ends were removed. Next, the spliced sequences were compared in the NCBI database (blast.ncbi.nlm.nih.gov) with the standard strains’ rRNA type strains/16S_ribosomal_RNA database. After that, the species with the highest homology was selected and an evolutionary tree was constructed to confirm that the strain was *P. stutzeri*.

### 3.2. Growth Characteristics of P. stutzeri and SRB

#### 3.2.1. Effect of Temperature on the Growth of the Strain

**Figure 3:**
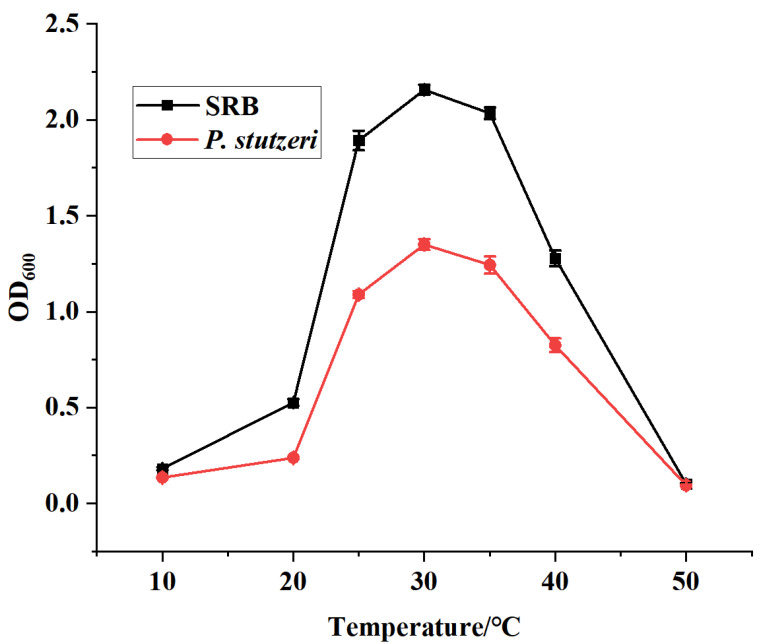
Effect of temperature on strain growth.

Activated SRB and *P. stutzeri* were inoculated into liquid medium at pH 7 and incubated at a temperature ranging from 10 to 50 °C. After 7 days, the OD_600_ values of the bacterial broths were measured and the results are shown in Figure 3.

From Figure 3, it can be seen that the isolated and purified strains of SRB and *P. stutzeri* maintained high bacterial concentrations in the range of 25–40 °C. The absorbance of both SRB and *P. stutzeri* strains reached the maximum at around 30 °C. In this experiment, the optimum growth temperature of both SRB and *P. stutzeri* was around 30 °C. Thus, temperature affected the growth of SRB and *P. stutzeri* to a similar extent.

#### 3.2.2. Effect of pH on the Growth of the Strain

**Figure 4:**
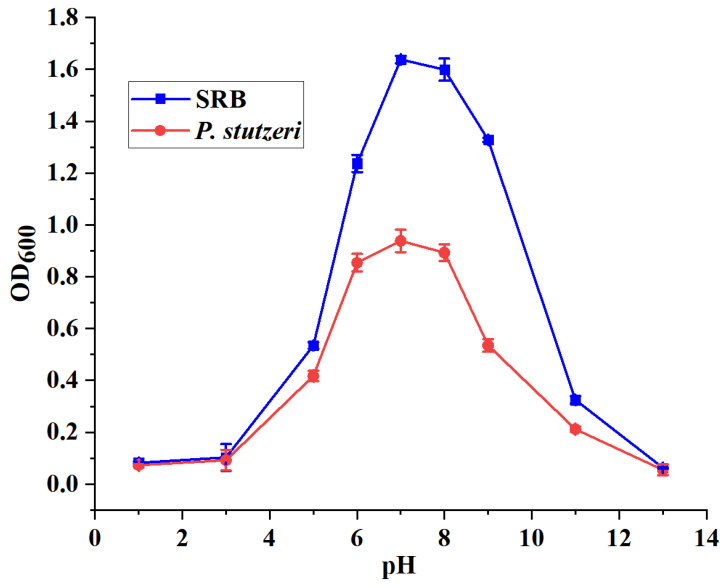
Effect of pH on strain growth.

After the activation, SRB and *P. stutzeri* were respectively inoculated into a liquid medium (the incubation temperature was 30 °C) with pH ranging from 1 to 13 to determine the effect of pH on the growth of SRB and *P. stutzeri*. OD_600_ values of the bacterial solution were measured after 7 days, and the results are shown in Figure 4.

As shown in Figure 4, the maximum concentration of both SRB and *P. stutzeri* was reached at pH 7. The experiment showed that both SRB and *P. stutzeri* were able to grow in a wide pH range from 5 to 8, and the optimum growth pH was about 7. Thus, the effect of environmental pH on the growth of SRB and *P. stutzeri* was similar. The above experiments provide a feasible basis for the coexistence of SRB and *P. stutzeri* in the same growth environment.

#### 3.2.3. Graphical Growth Curves of *P. stutzeri*, SRB and Co-Culture of *P. stutzeri* + SRB

**Figure 5:**
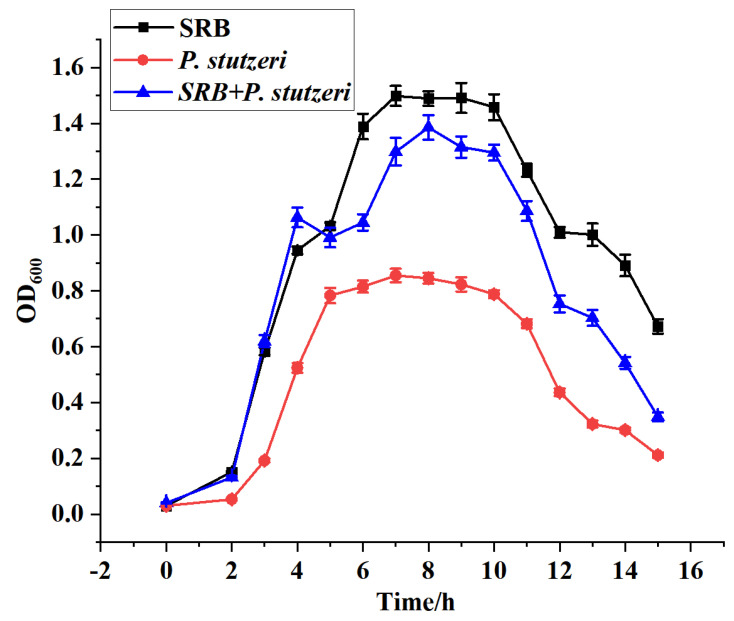
Growth curves of SRB, *P. stutzeri* and co-culture of *P. stutzeri* and SRB.

First, it was necessary to separately prepare the media of *P. stutzeri*, SRB, and mixed medium (mixed in the ratio of 1:1). Then, *P. stutzeri*, SRB and SRB + *P. stutzeri* mixture were inoculated into the above media at a 1/100 ratio and incubated in blue-capped culture flasks under anaerobic conditions at 30 °C constant temperature. After two days, samples were taken every 24 h and analyzed for OD_600_ values. Based on the results, the growth curves of SRB, *P. stutzeri* and co-culture of *P. stutzeri* + SRB were plotted, and the results are shown in Figure 5.

From Figure 5, it can be seen that SRB reached the logarithmic growth phase on the third day and entered the stable phase after 8 days. However, after 12 days, SRB gradually began to enter the decay phase due to nutrient depletion, which changed the optimal growth environment of SRB. In contrast, *P. stutzeri* entered the stable phase after 5 days and slowly started to enter the decay phase after 11 days. As a result, it can be concluded that the growth cycles of SRB and *P. stutzeri* are consistent.

When SRB was co-cultured with *P. stutzeri*, the growth of SRB was not inhibited by *P. stutzeri* in the first 2 days; the concentrations of the mixed bacteria on the third and fourth days were greater than those of the two bacteria in separate cultures; after the fifth day, the concentrations of the two bacteria in co-culture were higher than those of *P. stutzeri*, but lower than those of SRB in separate cultures. It can be tentatively assumed that the growth of SRB was inhibited by *P. stutzeri* when the two bacteria were co-cultured together. This is likely because when the two bacteria were co-cultured in the same medium, they grew competitively as the medium substrate was consumed over a longer incubation time. Namely, possible factors inhibiting the growth of SRB by *P. stutzeri* include their substrate utilization or their secretions.

### 3.3. Corrosion Inhibition of X70 Pipeline Steel

#### 3.3.1. Effect of Temperature on the Inhibition of SRB Corrosion by *P. stutzeri*

**Figure 6:**
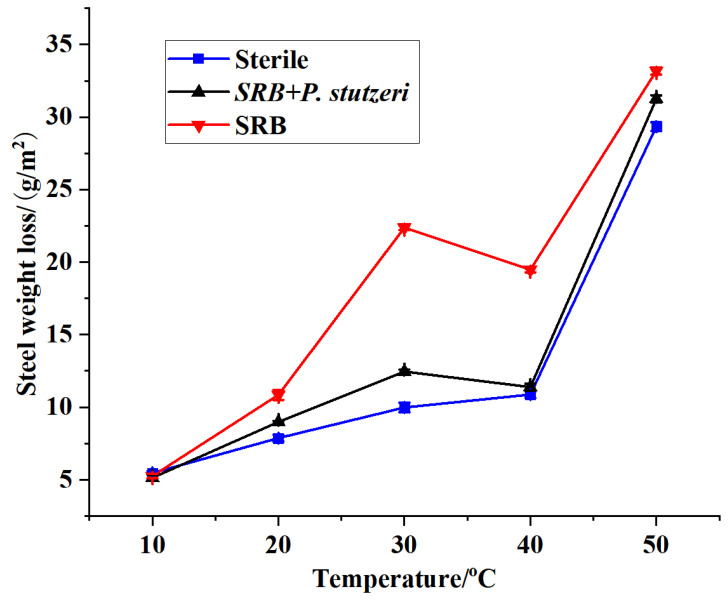
Weight loss curves (7 days) of X70 steel under different temperature conditions.

With the temperature change, there was a fairly obvious change in the weight loss of the samples, and the results are shown in Figure 6.

The weight loss was relatively low when the temperature was between 10 °C and 20 °C. With the increase in temperature, the weight loss increased significantly. The corrosion of the steel sheets was more severe in the system in which only SRB was inoculated at 30 °C, while at 50 °C severe corrosion was noted in all three systems. From the analysis, the main factors were identified: most of the microorganisms had a suitable growth temperature of 20–35 °C—the higher the temperature, the stronger the microbial activity. If the external temperature were reduced or increased, the growth of the microorganisms would be affected to a certain extent. At temperatures lower than 20 °C, the metabolic activity of microorganisms (e.g., related enzymes) would be inhibited; at temperatures as low as 10 °C, the growth rate of microorganisms would be significantly reduced and they would basically be in a dormant state, making the corrosion of X70 steel relatively weak. The corrosion under these conditions would mainly be based on electrochemical corrosion, and since the temperature is low, the electrochemical corrosion would also be relatively low. With the increase in temperature, the activity of bacteria increased. Since the weight loss of steel sheets in the medium only inoculated with SRB was more apparent between 30 and 40 °C, it can be concluded that SRB grew faster and were more active. 

Meanwhile, there was a slight trend of increased weight loss of steel sheets in the blank medium, mainly due to electrochemical corrosion. In the system inoculated with *P. stutzeri* + SRB, the weight loss of steel sheets was greatly reduced compared with the system inoculated with SRB only, indicating that *P. stutzeri* reduced the corrosion of X70 steel by SRB. When the temperature reached 40 °C, the growth of bacteria and the activity of enzymes would be somewhat inhibited by the high temperature. Hence, the corrosion induced by SRB was weakened, and the electrochemical corrosion was enhanced. Moreover, when the temperature reached 50 °C, as the temperature was far too high, the activity of bacteria was reduced by the influence of temperature. However, this high temperature promoted electrochemical corrosion, which caused more severe corrosion and a greater loss of weight. Thus, it can be concluded that *P. stutzeri* may have a good inhibitory effect on the corrosion of steel sheets caused by SRB in a wide temperature range.

#### 3.3.2. Effect of Time on the Inhibition of SRB Corrosion by *P. stutzeri*

**Figure 7:**
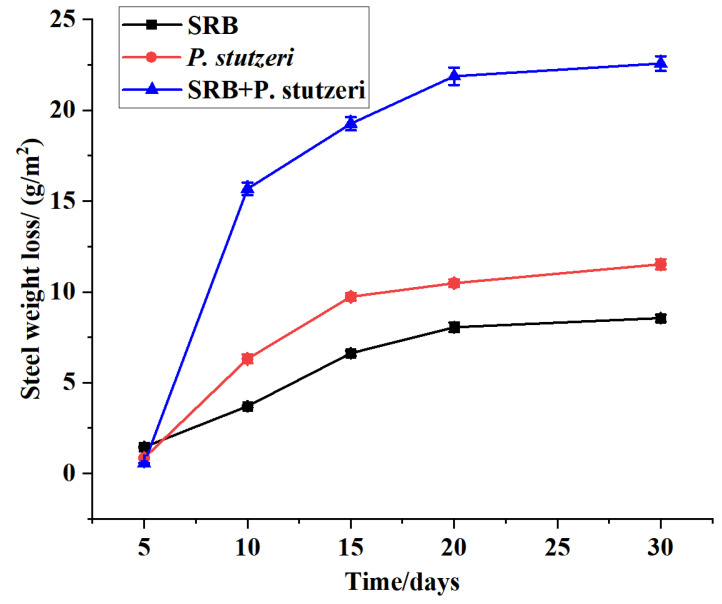
Weight loss curves of X70 steel under different test times.

There was a gradual weight loss per area of the steel sheets as the incubation time increased (the incubation temperature was 30 °C), as shown in Figure 7.

The corrosion loss of the steel sheets inoculated with SRB was much greater than that observed in the other two tested systems. The 30-day loss was 8.57 g/m^2^, measured from the steel sheets in the blank group, which was mainly due to electrochemical corrosion caused by certain salts in the incubation medium. The corrosion rate of X70 steel in the test system inoculated with SRB alone was greater, which reached 15.67 g/m^2^ at 10 days, 19.26 g/m^2^ at 15 days, 21.87 g/m^2^ at 20 days and 22.57 g/m^2^ at 30 days. The growth curve of SRB bacteria showed four typical phases: retardation phase (0–2 days), log phase (3–6 days), stabilization phase (7–11 days) and decay phase (>12 days). In the range of 0–5 days, SRB were in the activation period after inoculation, and the number of active bacteria was small. Therefore, the corrosion was mainly caused by chemical corrosion; in days 5–10, the number of SRB increased drastically, and the corrosion enhanced. Notably, after 7 days, the number of strains reached the maximum, the number of active bacteria was high, both enzyme activation and metabolism were at the peak, and the corrosion also intensified. Although the number of bacteria remained at a high state in the period of 10–15 days, the weight loss was lower than that in the 5–10 days period, probably because the growth of high quantities of bacteria led to the formation of biofilms on the surface of the steel sheets, which slowed the rate of increase of corrosion. Furthermore, at 20–30 days, corrosion was significantly delayed due to the large consumption of nutrients at this stage, while the toxic metabolites accumulated in large quantities, leading to an increase in SRB mortality and a decrease in viable bacteria. At the same time, the chemical corrosion also slowed because inorganic substances were consumed during their growth. In that case, it can also play a role in mitigating the corrosion on the surface layer as the corrosion product film became thicker and its combination with the microbial film was denser, which led to the corrosion into a slower stage after 15 days. The weight loss of steel sheets in the medium inoculated with SRB + *P. stutzeri* was 11.52 g/m^2^ after 30 d immersion experiment. During the 0–30 days period, the weight loss in the mixed bacteria culture was significantly lower than that of SRB alone; between 15 and 30 days, the weight loss was basically at a stable stage. It was assumed that in the co-culture of *P. stutzeri* + SRB, the presence of *P. stutzeri* influenced the growth of SRB and slowed the corrosion of steel sheets by SRB, while on the other hand, the bacteria, extracellular polymers and corrosion products were mixed to generate a dense biofilm, which played a protective role on the steel sheets. Thus, it can be noted that *P. stutzeri* has a better protective effect on the corrosion of steel sheets caused by SRB with the extension of time.

#### 3.3.3. Effect of Initial pH on the Inhibition of SRB Corrosion by *P. stutzeri*

**Figure 8:**
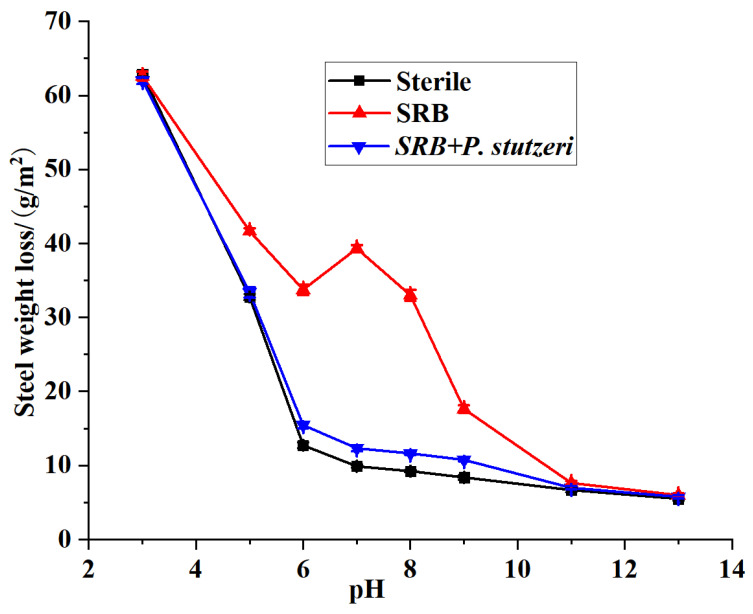
Weight loss curves of X70 steel under different pH.

The rules of electrochemical corrosion of steel affected by pH were as follows: when pH < 4, the corrosion of carbon steel was severe, mainly caused by hydrogen evolution corrosion; at pH 5–13, the corrosion was slower, mainly caused by oxygen absorption corrosion. From Figure 8, it can be seen that when pH 3, steel corrosion was more serious. At this stage, the role of microorganisms was not prominent; the bacteria in the acidic medium grew slowly in general, so it the main corrosion mechanism was chemical hydrogen precipitation corrosion due to acidic conditions, and the rate of X70 corrosion was higher. With increased pH, electrochemical corrosion weakened, and microbial corrosion increased. At pH 6–8, conditions were more suitable for microbial growth, and SRB and chemistry caused the corrosion. At pH 7, the corrosion caused by SRB was more severe, and the weight loss reached 39.31 g/m^2^, but the weight loss in the combined system of *P. stutzeri* + SRB was weakened to 12.33 g/m^2^; meanwhile, in the blank medium system, the weight loss was 9.89 g/m^2^, which was mainly due to chemical oxygen absorption corrosion. Discounting the electrochemical corrosion, the addition of *P. stutzeri* resulted in an 83% reduction in weight loss. Therefore, *P. stutzeri* provided better protection against steel sheet corrosion at pH 7. At pH > 9, the corrosion was relatively weaker because both chemical and microbial corrosion were slowed down under alkaline conditions. Overall, the growth of microorganisms was somewhat restricted in alkaline environments, enzyme activity was reduced, and there was a relative decrease in both corrosion and corrosion resistance. Li et al. also showed that in an alkaline environment, a thick oxide film was generated on the surface layer of pipeline steel, which acted as a passivation layer and slowed the corrosion rate [35]. Therefore, the protection of *P. stutzeri* against SRB corrosion was better in the pH 6–8 environment.

#### 3.3.4. Analysis of Elements in Corrosion Products

**Figure 9:**
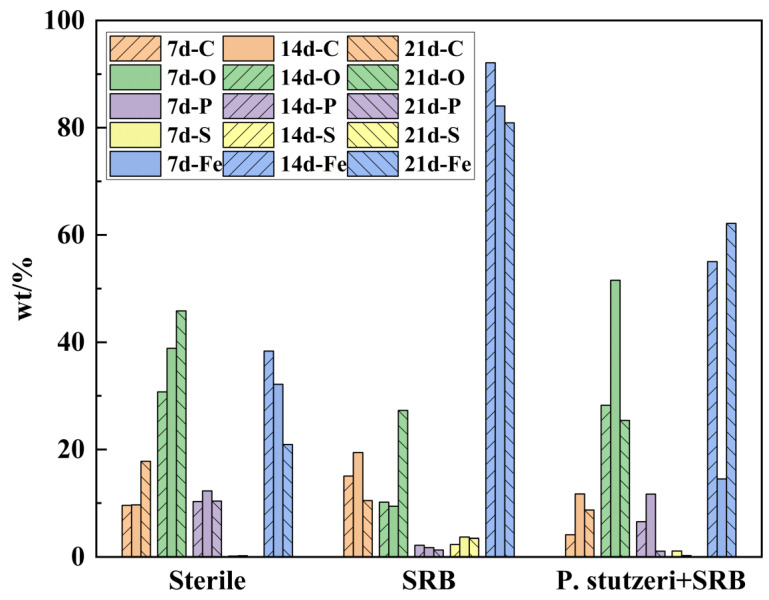
Element content of corrosion products on steel coupon surface.

From the analysis of corrosion products (Figure 9), it can be seen that the corrosion products in the sterile environment were mainly with inorganic compounds, such as iron oxides, and the P elements were mainly derived from K_2_HPO_4_ in the medium. The content of Fe and S in the corrosion products inoculated with SRB was significantly higher than that in the sterile environment and in the environment with *P. stutzeri*; presumably, the corrosion products mainly contained sulfides and iron oxides. In the environment with mixed bacteria, the corrosion products mainly contained C, O, Fe and S, but the content of S was significantly lower than that of corrosion products inoculated with SRB. The oxidation of sulfatide induced by *P. stutzeri* hindered the accumulation of corrosive sulfide, and the SRB-involved corrosion can be weakened thereby.

#### 3.3.5. Tafel Curve

The polarization curve test is damaging, and hence it is also known as a disposable test, where irreversible damage is caused to the sample surface during the measurement. Because the Tafel polarization can damage the sample surface, which can effectively exclude interference, the relationship between the polarization current and electrode potential of the respective anodic and cathodic reactions can be observed separately, rendering the polarization curves capable of revealing the mechanism of electrochemical reactions and their kinetic characteristics in depth.

**Figure 10:**
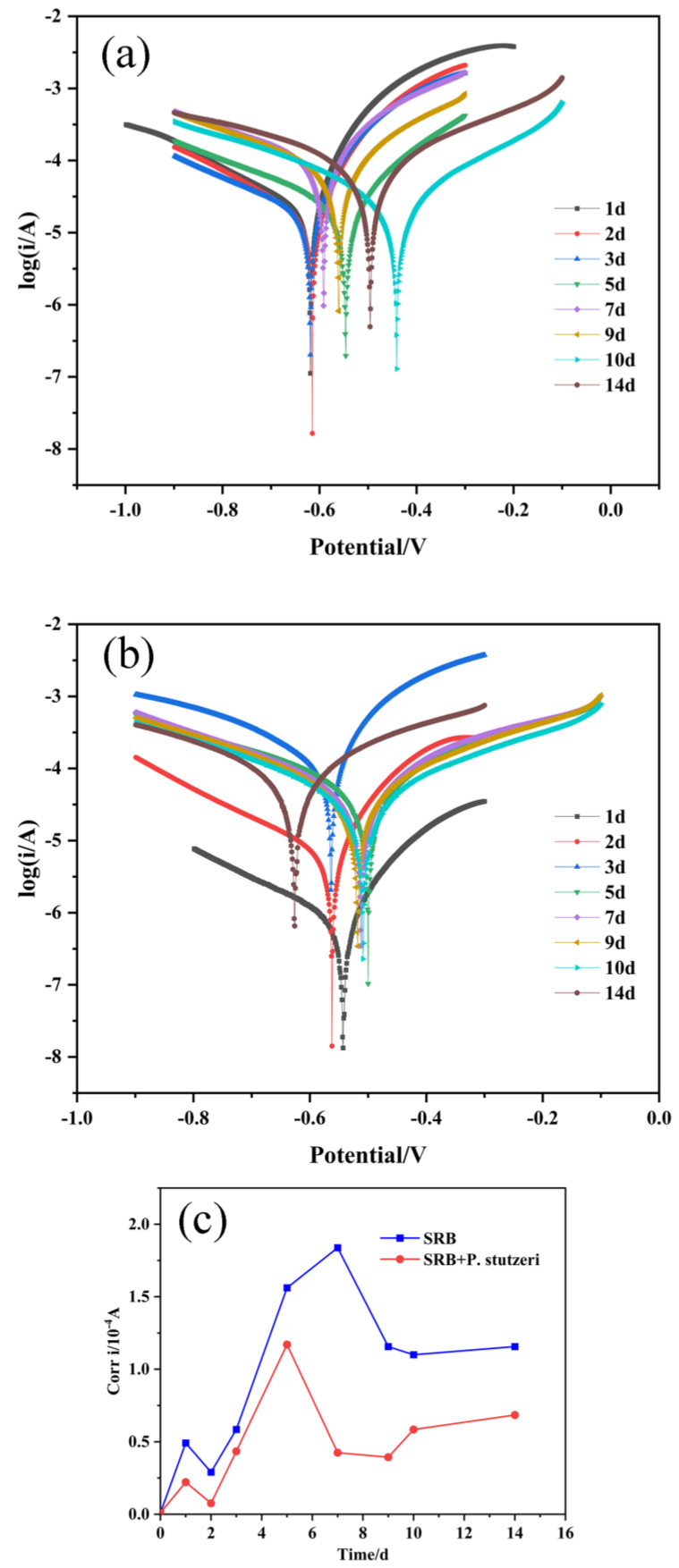
(**a**) The potentiodynamic polarization curves of SRB; (**b**) the potentiodynamic polarization curves of SRB + *P. stutzeri*; (**c**) the corrosion current density.

The polarization curves of X70 steel samples in SRB and SRB + *P. stutzeri* solutions from 0 to 14 days of inoculation were measured with activated strains. In addition, the corrosion current density (i_corr_) was obtained after fitting using extrapolation software (Origin 2018), and the variation trend is shown in Figure 10.

Figure 10a,b are the corrosion polarization curves for SRB and SRB + *P. stutzeri* samples, respectively, and Figure 10c shows the corrosion current density in both media. As can be seen from Figure 10, the corrosion current density in both media was the same at the beginning of the experiment, and with the extension of the incubation time, the corrosion current density of X70 steel in the media inoculated with SRB only reached the maximum at 7 days. At this stage, the growth rate of SRB was faster and the number of strains increased, while corrosion intensified. In the next 7 days, there was a decrease in corrosion current density compared to the seventh day owing to the formation of corrosion products on the surface, which had a protective effect on the specimens. As can be seen from the test results of 14 days of inoculation, after 2 days, the corrosion current density of X70 steel in the mixed medium of SRB + *P. stutzeri* was always lower than that of the medium inoculated with SRB alone, which indicated that the addition of *P. stutzeri* had a certain inhibitory effect on the corrosion caused by SRB. In brief, the corrosion current density generally showed a trend of first increasing and then decreasing, which means the corrosion was initially enhanced and then weakened.

#### 3.3.6. Long-Term Exposure Weight Loss Test

**Figure 11:**
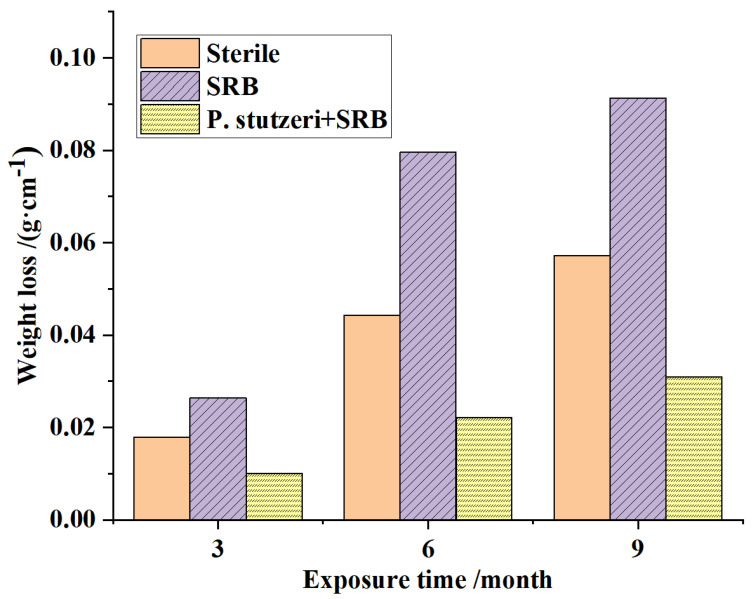
Weight losses of steel coupons from long-term exposure (3, 6, 9 months).

The weight losses of X70 steel sheets exposed to media for 3, 6 and 9 months are shown in Figure 11.

The weight loss of the steel sheets in all systems gradually increased with time, which indicates that the degree of corrosion varied in different environments. Still, as shown in Figure 11, the media inoculated with SRB alone showed more weight loss and more severe corrosion. The weight of specimens in the blank group lost slightly more than those inoculated with SRB + *P. stutzeri* because the blank medium had a higher electrolyte concentration than the solution inoculated with SRB + *P. stutzeri*, where bacteria grew and consumed part of the electrolytes. This is consistent with the results of the short-time corrosion weight loss experiments described above. In addition, the weight loss of steel sheets in the media inoculated with SRB + *P. stutzeri* was significantly lower than that in the system inoculated with SRB, which suggests that *P. stutzeri* had a better effect on preventing and controlling SRB-induced corrosion on steel sheets.

## 4. Conclusions

(1) A strain of *P. stutzeri* was obtained by isolation and purification. The strain is a Gram-negative bacterium with short rod-shaped cells, about 0.5–1 µm in length, and white transparent colonies, which could discolor Giltay medium and produce gas, showing that it has denitrification ability. 

(2) The sequencing results of 16SrDNA also verified the properties of the *P. stutzeri* strain: its growth conditions were similar to those of SRB, with an optimum culture temperature of about 30 °C and an optimum growth pH of about 7. It entered the stabilization phase after 5 days and started to enter the decay phase slowly after 11 days. The growth cycle was the same as that of SRB, and the two bacteria can co-exist in the common growth environment.

(3) The results of the weight loss tests showed that *P. stutzeri* could inhibit the corrosion of X70 steel caused by SRB at 20–40 °C and pH 6–8. The electrochemical results showed that SRB promoted the corrosion of X70 steel, and the corrosion of X70 steel was inhibited in the *P. stutzeri* + SRB media. In addition, the corrosion current density of X70 steel in the media containing mixed bacteria was less than that of the environment inoculated with SRB alone, and the EDS results showed that the elemental S content was significantly lower than that of the corrosion products inoculated with SRB. The results of the weight loss tests also showed that *P. stutzeri* had a better inhibitory effect on the corrosion of X70 steel caused by SRB in the medium at 3, 6 and 9 months.

## Figures and Tables

**Table 1:** The inocula volumes of the three experimental groups.

	Control (mL)	SRB (mL)	*P. stutzeri* + SRB (mL)
Sterile water	5	----	----
SRB	----	5	5
*P. stutzeri*	----	----	5

## Data Availability

Not applicable.
